# Greenhouse gas flux with reflooding of a drained salt marsh soil

**DOI:** 10.7717/peerj.5659

**Published:** 2018-11-15

**Authors:** Jan T. Wollenberg, Asim Biswas, Gail L. Chmura

**Affiliations:** 1 Department of Geography, McGill University, Montreal, Quebec, Canada; 2 School of Environmental Sciences, University of Guelph, Guelph, Ontario, Canada

**Keywords:** Greenhouse gas flux, Methane, Nitrous oxide, Salt marsh, Agricultural marsh, Managed realignment, Blue carbon

## Abstract

Salt marshes are highly effective carbon (C) sinks and bury more C per square meter annually than any other ecosystem. Reclamation and anthropogenic impacts, however, have resulted in extensive losses of salt marshes. Carbon credits can be generated and sold by restoring marshes, but only if C sequestration and net reductions in greenhouse gases (GHG) are reliably quantified. Restored marshes, however, may exhibit different patterns of GHG emissions than natural marshes and it is possible that they could temporarily become sources of N_2_O even in the usually N-limited estuarine environment. Research on short-term GHG flux following salt marsh restoration is limited to studies of two restored marshes which examined GHG flux more than six months after the return of tidal flooding. Here we report on a laboratory experiment in which soil cores collected from a drained agricultural marsh on the St. Lawrence Estuary were flooded with estuary water. Gas flux measurements immediately after flooding revealed small increases in N_2_O and CH_4_, but a large decline in CO_2_ yielding, from a climatic perspective, a net cooling effect over the observation period. In addition to restoring the land’s capacity to sequester C once a marsh develops, returning tidal flooding thus appears to have the added benefit of stemming large ongoing C losses. With more than 400 km^2^ of undeveloped dykeland, Eastern Canada is well positioned to restore large sections of marsh and contribute to reducing atmospheric CO_2_ concentrations.

## Introduction

Salt marshes provide valuable ecosystem services including habitat provision and storm protection (e.g., Chmura & Burdick, 2012; Barbier et al., 2013). In addition, salt marshes have been recognized for their value as highly effective carbon (C) sinks (Chmura et al., 2003; Mcleod et al., 2011) and the term “blue carbon” has been coined for the C stored in salt marshes, mangroves and seagrass beds (Nellemann et al., 2009). Carbon dioxide (CO_2_) is fixed by marsh vegetation through photosynthesis and stored in both aboveground and belowground biomass. The persistence of anaerobic conditions results in low rates of decomposition (Kristensen, Ahmed & Devol, 1995; Kirwan & Blum, 2011) and over time salt marshes accrete both vertically and laterally with the accumulation of organic matter and sediment delivered by tidal floodwaters (Redfield, 1965; Fitzgerald et al., 2008). Unlike freshwater wetlands which can be significant sources of CH_4_ (Bridgham et al., 2013), salt marshes are typically negligible sources of CH_4_ and N_2_O. The former is due to the presence of abundant sulfate in marsh soils that hinders CH_4_ production (Poffenbarger, Needelman & Megonigal, 2011). Where the supply of N as NO_3_^−^ is minimal, N_2_O emissions are also negligible (Chmura, Kellman & Guntenspergen, 2011).

Over the past 50 to 100 years, human activities including land reclamation through drainage have resulted in the loss of 25–50% of salt marsh area globally (Adam, 2002; Pendleton et al., 2012). On Canada’s St. Lawrence River estuary, 135 km^2^ (63%) of pre-existing marsh has been drained (Chmura et al., 2014). Once drainage infrastructure is in place, the water table of the reclaimed marsh area recedes causing the decay of organic matter to accelerate as the formerly saturated soil is aerated (Portnoy, 1999). Net C losses are thus likely over time as the land subsides (Drexler, De Fontaine & Deveral, 2009; Pendleton et al., 2012) and the former marsh soil loses its capacity to act as a C sink; losses as high as 80% have been reported for drained marshes in the San Joaquin Delta in California (Drexler, De Fontaine & Deveral, 2009).

Yet while the global decline of this important C sink continues today, in certain countries this trend has now been reversed with efforts to restore salt marshes through a process known as managed realignment (MR) (French, 2006; Pendleton et al., 2012). The process of MR involves the landward movement, breaching, or removal of a sea defence structure to restore tidal influence and promote the creation of new intertidal habitat (French, 2006). Managed realignment projects have demonstrated that even with relatively limited management or pre-treatment, allowing tidal flow to return to a drained and low-lying agricultural field will quickly produce new habitat dominated by salt marsh plants (French et al., 2000; Wolters, Garbutt & Bakker, 2005; Burden et al., 2013).

Although restoring marsh habitat can help reduce atmospheric concentrations of CO_2_, under some conditions salt marshes can be sources of N_2_O and CH_4_. In marshes with elevated nutrient inputs, for example, N_2_O emission can be substantial and could reduce the value of soil C storage from the perspective of climate change mitigation (Moseman-Valtierra et al., 2011; Chmura et al., 2016; Roughan et al., 2018). Roughan et al. (2018) observed significant N_2_O emissions from Prince Edward Island marshes in watersheds subject to N runoff from intensively farmed watersheds. N_2_O flux is of particular concern as it has a global warming potential (GWP) 265 times that of CO_2_ over a 100-year time horizon (Myhre et al., 2013). When extrapolated to the entire growing season with the sustained-flux global warming/cooling potential (SGWP/SGCP) developed by Neubauer & Megonigal (2015), the N_2_O flux offset ∼20% of the average annual C sequestration of the marshes.

Restored agricultural marshes, however, may exhibit patterns of GHG emissions that differ from natural marshes. Since agricultural management often includes application of manure or inorganic fertilizer they could temporarily become sources of N_2_O even in the usually N-limited estuarine environment. To investigate these differences in GHG dynamics, Blackwell, Yamulki & Bol (2010) experimentally flooded soil cores collected from a MR marsh approximately 6 months after MR and a natural marsh in the upper estuary of the River Torridge, UK. Prior to the realignment the MR site consisted of abandoned agricultural land reclaimed from the salt marsh approximately 200 years ago (Blackwell, Yamulki & Bol, 2010). In contrast to the natural marsh, they found that the soil cores from the formerly drained MR had N_2_O emission even when floodwater was N limited, presumably due to an intrinsic source of NO_3_^−^. Similarly, Adams, Andrews & Jickells (2012) studied the rates of C burial and GHG emissions (CO_2_, N_2_O and CH_4_) in natural and MR salt marshes of the Blackwater estuary, UK and found that GHG emissions, particularly N_2_O, were highest in the MR marshes. The highest N_2_O emissions observed originated from an MR site formerly used as pasture which also had the highest pre-breach soil N content (mean of 0.94 wt. % N) of the five MR sites. Due to its large global warming potential (GWP), N_2_O flux reduced the net cooling effect of C sequestration by as much as 49% in some of the MR marshes investigated (Adams, Andrews & Jickells, 2012).

Previous studies have shown that MR and soil flooding can result in rapid changes in soils of recovering marshes (e.g., Portnoy & Giblin, 1997; Blackwell, Hogan & Maltby, 2004) which can contribute to the multiple pathways for production of N_2_O (Kool et al., 2011). Portnoy & Giblin (1997) flooded soil cores from a historically drained salt marsh on Cape Cod and found a large NH_4_^+^-N mobilization immediately after re-flooding which resulted in a 40 to 60-fold increase in porewater NH_4_^+^-N concentration. Similar research following the MR of abandoned agricultural lands in the River Torridge estuary, UK, revealed that MR quickly transformed the site’s topsoil (0–10 cm) from an oxidized to a reducing environment and increased topsoil pore water NH_4_^+^-N concentrations from 0.1 to 10.1 mg L^−1^ (Blackwell, Hogan & Maltby, 2004). Studies employing stable isotopes have provided evidence that ammonia oxidizing bacteria can significantly contribute to soil N_2_O emissions in flooded soil (Baggs, 2011; Kool et al., 2011).

Examining the impact of flooding would require sampling at high tide which presents logistical challenges. Static chamber measurements are thus often taken during low tide when nutrient concentrations and N_2_O fluxes are likely to be lower (Adams, Andrews & Jickells, 2012). Roughan et al. (2018) found N_2_O flux varied with the tidal cycle and observed the highest N_2_O fluxes immediately after the high tide receded. Simply using an average of high tide and low tide fluxes extrapolated over 24 hours may thus overestimate or underestimate daily N_2_O fluxes (Roughan et al., 2018). To accurately model the soil’s response to flooding, it is important to verify the effect of flooding on N_2_O emissions at high tide, low tide, and mid tide (halfway between high tide and low tide).

Although restoration of salt marsh can mitigate climate change, considerable gaps in our knowledge with regards to GHG dynamics in recovering marshes remain, particularly immediately after estuary water is returned (Kroeger et al., 2017). Soil core incubation experiments are an alternative to field sampling given the logistical challenges of capturing emissions at the exact moment of re-flooding. The purpose of our study is to assess the GHG flux of the soil immediately after flooding. This data will help to establish a baseline for evaluating the potential changes in GHG flux following the restoration of estuarine flooding to dykelands.

## Materials and Methods

### Study site

In the Kamouraska region, which spans from Saint-Roch-des Aulnaies to Trois-Pistoles on the southern shore of the St. Lawrence River, the drained marsh soils are acid sulfate soils belonging to the de l’Anse series in Quebec (Dekimpe, Laverdiere & Baril, 1988). Surface soils (0–35 cm) are composed primarily of silty clay (Dekimpe, Laverdiere & Baril, 1988).

**Figure 1:**
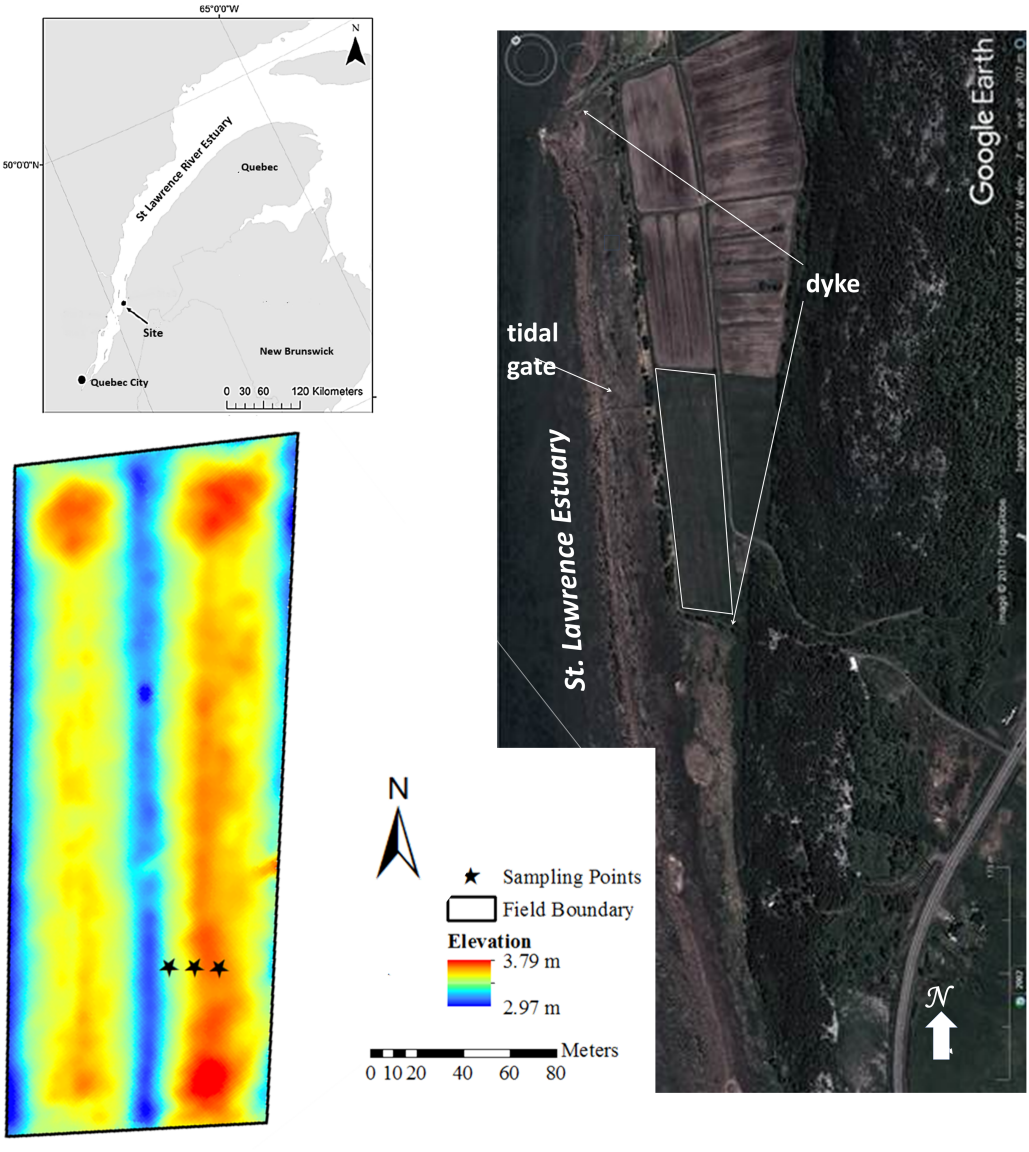
Study site and digital elevation model located in Quebec’s Kamouraska region, Canada (adapted from Van Ardenne, 2016). Map Data: Google/DigitalGlobe.

Soil cores were collected from a farm on a drained and dyked salt marsh (47°41′36.54″N, 69°42′38.95″W) located in the Kamouraska region (Fig. 1).

**Figure 2:**
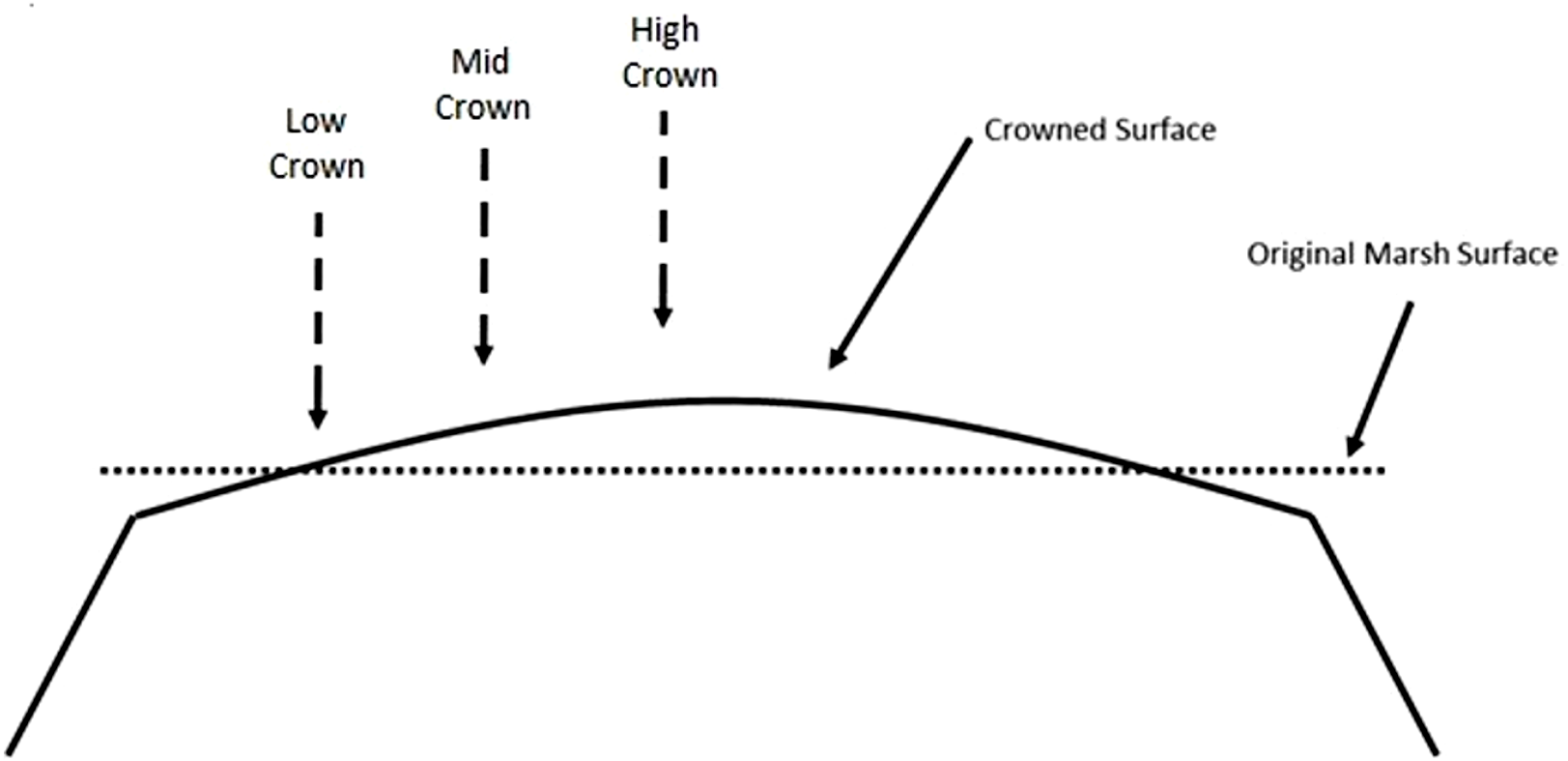
Diagram of a crowned farm field and approximate location of sampling points (Adapted from Van Ardenne, 2016).

The dyke was constructed in 1986 (A Parent, pers. comm., 2016) and conventional drainage infrastructure was employed. Several drainage ditches approximately 1 to 2 m deep allow runoff from the upland and field to drain into the river. Tidal gates were installed where the ditches intersect the dyke to prevent the inflow of tidal water at high tide and allow water from the farm and the upland to drain out to the river at low tide. The site was also subject to an additional modification known as crowning, or landforming, which involves transferring the soil adjacent to the ditches to the mid-ditch area, creating a crown and a slope from the crown towards the ditches to promote drainage (Fig. 2).

The farmland is used to grow alternating grain and hay crops in mixed rotation (A Parent, pers. comm., 2016). The field is plowed to a depth of approximately 30 cm at which a plow pan is found. The reduced permeability of the compacted plow pan slows the percolation of water at depth causing water to drain horizontally into the adjacent ditches.

The Kamouraska region has a cool and wet climate with a yearly annual average temperature of 4.5 °C (Environment Canada, 2016). The daily average temperature in July is 19 °C and the daily average maximum in January is −12 °C (Environment Canada, 2016). The soil freezes in mid to late November and thaws in late April to early May. The region receives an annual average of 1,140 mm precipitation (Environment Canada, 2016). Tidal amplitudes at nearby Riviere-du-Loup are between 3.5 and 5.6 m (Fisheries and Oceans Canada, 2015).

### Field sampling

Soil cores were collected at three elevations within the field: at the low-crown, mid and crown top (Fig. 2). The first location (low-crown) was placed near the drainage ditch and represents the section of the field from which soil was removed during crowning modifications. The second location (mid-crown) was selected approximately 25 m towards the upland edge between the ditch and the center of the field; this location was chosen to represent an area which was less likely to have had any soil added or removed during crowning modifications. The third location (high-crown) was placed near the center of the field to represent the top of the crowned area where soil was added.

The cores were collected on October 31, 2015. Five replicate cores were collected from low, mid and high crown locations by driving 40 cm sections of a 10 cm-diameter HDPE pipe into the soil using a truck-mounted hydraulic borer. The pipes were driven approximately 30 cm into the soil, or until refusal, and the cores were then dug out by hand. A denser and more compacted soil was encountered at the mid-crown and high-crown locations; cores collected from these locations were thus subject to compaction as the pipes were driven into the soil. The mean % compaction (calculated from the length of the outside and inside of the core after insertion into soil) measured for cores collected in the low-crown, mid-crown, and high-crown locations were 3.9% (*n* = 5), 16.5% (*n* = 5), and 26.6% (*n* = 5), respectively. Cores were sealed at each end using fitted HDPE caps and stored under refrigeration.

A hole was dug adjacent to the cores at each of the three locations in the field to collect bulk soil samples for NH_4_^+^ and NO_3_^−^ analysis. Three samples were collected from the wall of each hole from depths of 0–10 cm, 10–20 cm, and 20–30 cm. At high tide 32 L of estuary water (salinity 9 psu) was collected near La Pocatière approximately 50 km upstream of the study site. The water was frozen following collection.

### Laboratory analyses

Soil cores previously collected across the farm field by Van Ardenne (2016) were used to determine surface soil (0–30 cm) bulk density and organic C content. All samples were oven dried at 60 °C until there was no additional mass lost. Samples were then ground using a mortar and pestle. Representative sub-samples of ∼2.5 g were placed in crucibles and dried at 60 °C until all moisture was lost and weighed in preparation for loss on ignition (LOI). In a programmable muffle furnace, the sub-samples were heated to 350 °C for 1 hour followed by 4 hours at 550 °C (Heiri, Lotter & Lemcke, 2001). Samples were removed from the muffle furnace and placed in a desiccator to cool to room temperature prior to weighing. If the difference in mass loss between the two replicate samples exceeded 10% additional replicates were run. The organic C content of each sample was estimated using a conversion factor developed by Craft, Seneca & Broome (1991): Organic C fraction = 0.40(LOI fraction) + (0.025 * LOI fraction)^2^. The NH_4_^+^ and NO_3_^−^ content were determined using potassium chloride extraction and the automated colorimetric method (Maynard & Kalra, 1993).

To prepare cores for the gas flux experiment, holes were drilled in the center of each bottom cap and through the wall of the core tube approximately 1 mm above the soil surface. Clear plastic tubing was inserted into the holes and sealed in place with silicon caulking to allow both surface water and percolating water to drain after each flooding cycle. The bottom caps of the soil cores were lined with cheese cloth, 20 µm filter paper, and a thin layer of fine gravel to prevent the loss of soil as water drained. A control was prepared using the same materials (river water, HDPE piping, silicon caulking, gravel, filter paper, and cheese cloth) to confirm that any changes in gas flux observed were due to microbial activity in the soil cores rather than the composition of the materials used in the experiment. Headspace sampling of river water in the control tube yielded negligible gas flux of just −0.014 µmol CH_4_ hr^−1^, −0.010 µmol N_2_O hr^−1^, and 20.89 µmol CO_2_ hr^−1^. Changes observed during soil flooding were thus attributable to differences in soil microbial processes as the influence of the different materials were negligible. Gas concentrations measured in the ambient air in the lab at the beginning of the experiment were 0.331 ppm N_2_O, 1.991 ppm CH_4_, and 608.18 ppm CO_2_; concentrations of each gas were within the expected range based on current atmospheric GHG concentrations (Blasing, 2016).

Cores were frozen at −10 °C for one week prior to use to simulate the natural freezing that occurs in the region over winter. Following freezing, the cores were incubated at room temperature (ranging from 23 to 25 °C) for five days prior to beginning the experiment. Cores were flooded by gently pouring the estuary water over top of the soil column until 3 cm of standing water was present above the soil surface and the water level remained constant. The flooding pattern is modeled after that of Blackwell, Yamulki & Bol (2010) to simulate semi-diurnal tidal flooding.

A headspace was created above the soil core during each sampling event using a 10-cm diameter plastic cap fitted with a rubber septum for gas sampling. During each sampling event the core was capped to allow the GHG to accumulate in the headspace. After installing the cap, 15 mL gas samples were collected over the course of 1 hr at 20 min intervals (0, 20, 40, and 60 min). Each sample was collected through the septum using a gas tight syringe with a stopcock. Prior to sampling, the headspace was flushed by withdrawing and reinjecting 80 mL of air using the syringe to ensure proper mixing of the headspace (Wang, Cai & Yan, 2004).

Gas samples were collected at simulated low-tide, mid-tide, and high-tide periods during the first, second, and fifth day of flooding. All samples were analyzed on a Bruker GC 450 series gas chromatograph fitted with an electron capture detector (with carrier gas argon) and flame ionization detector (with carrier gas helium).

### Gas flux calculation

Flux calculations derived from changes in headspace concentrations are often estimated by applying a linear regression to the measurements (Koponen & Martikainen, 2004; Kroon et al., 2008). In certain cases, however, non-linearity in changes in headspace concentrations can lead to a poor-fit of a linear curve (Kroon et al., 2008; McVicar & Kellman, 2014). McVicar & Kellman (2014) compared results derived from fitting a slope through all the points to the fluxes calculated for each 20-min interval and then summed together to determine the total flux over 1 hr and found that fluxes were comparable. Gas flux was thus calculated using the summation method described by McVicar & Kellman (2014) based on the ideal gas law, differences in concentrations over time, the volume of the headspace, and the surface area of the soil core per the following equation.

\begin{eqnarray*}& & \mathrm{\mu }\mathrm{mol} {\mathrm{m}}^{-2}{\mathrm{hr}}^{-1}=[\text{final conc. ppm}-\text{initial conc. ppm})\times & & \quad [1,000 (\mathrm{L}{\mathrm{m}}^{-3})/[((22.7\times (\text{air temp}(\textdegree C)+273))/273)(\mathrm{L} {\mathrm{mol}}^{-1})]\times & & \quad \text{chamber volume}({\mathrm{m}}^{3})]/\text{collar area} ({\mathrm{m}}^{2}) \end{eqnarray*}

### Statistical analyses

The GWP has been used to compare the radiative forcing of different GHG (Myhre et al., 2013). In our study, we apply the sustained-flux global warming/cooling potential (SGWP/SGCP) developed by Neubauer & Megonigal (2015) (Table 1). The SGWP/SGCP metrics have been proposed as more representative for ecosystem studies. We used the SGWP and SGCP metrics over a 100-year time horizon to determine how flooding changed the climate role of the dyked soils; SGCP values were used for uptake (i.e., negative flux) and SGWP were used for emission (i.e., positive flux). The 100-year time horizon was chosen as C registries typically require that C offset projects be evaluated for a minimum period of 100 yr.

**Table 1:** Global Warming Potentials (GWPs), Sustained Global Warming Potentials (SGWP), and Sustained Global Cooling Potentials (SGCPs) from Neubauer & Megonigal (2015).

Gas	(Years)		(Emissions)	(Uptake)
	Time frame	GWP	SGWP	SGCP
CO_2_	any	1	1	1
CH_4_	20	87	96	153
	100	32	45	203
	500	11	14	288
N_2_O	20	260	250	264
	100	263	270	349
	500	132	181	491

Statistical analyses were performed using IBM SPSS statistics 22 (Armonk, NY, USA). Spatial and temporal differences in GHG fluxes were tested using one-way ANOVA. Pearson’s correlation was used to test the relationship between soil N content, % soil compaction, and N_2_O flux. A one-sample t-test was used to determine if the mean gas fluxes from the low, mid and high crown cores were significantly different from zero. The Anderson-Darling method revealed that flux data did not vary significantly from the normal distribution. The significance threshold for all analyses was set at 0.05.

## Results

Soil properties varied across the study site (Table 2).

**Table 2:** Soil properties for a drained salt marsh on the St. Lawrence estuary.

	Location	*n*	Mean	Standard deviation
Bulk Density (g cm^−3^)	All areas	30	0.7	0.18
% Organic C	All areas	30	6.3	3.7
NH_4_^+^ (mg kg^−1^)	Low crown	3	3.37	3.41
NO_3_^−^ (mg kg^−1^)	Low crown	3	0.82	0.86
NH_4_^+^ (mg kg^−1^)	Mid crown	3	1.45	0.34
NO_3_^−^ (mg kg^−1^)	Mid crown	3	0.35	0.1
NH_4_^+^ (mg kg^−1^)	High crown	3	3.28	0.97
NO_3_^−^ (mg kg^−1^)	High crown	3	0.49	0.17

### CO_2_ flux

**Figure 3:**
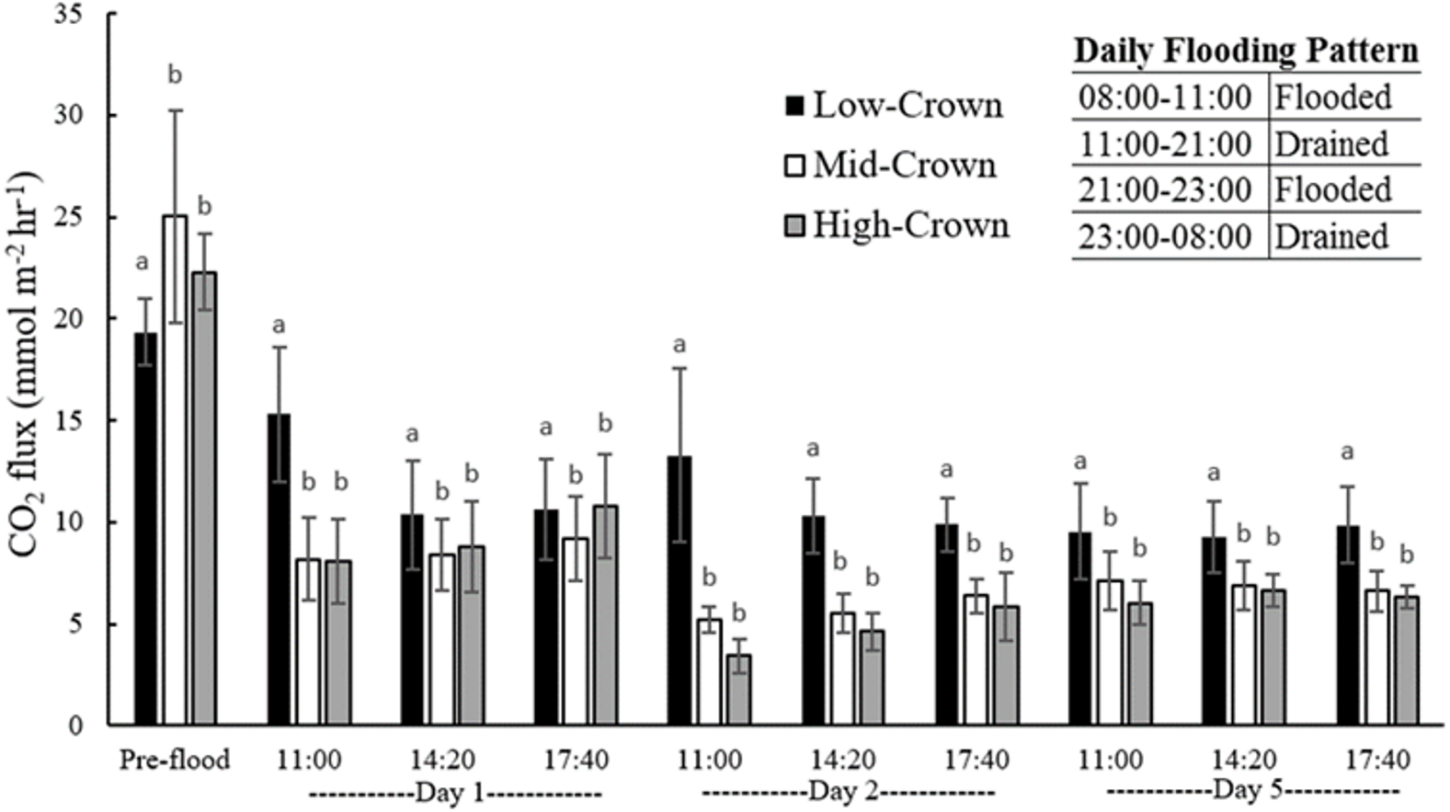
Mean CO_2_ flux from flooded soil cores collected from a drained salt marsh on the St. Lawrence estuary. CO_2_ flux was significantly higher in low-crown cores compared with both mid-crown (*p* < 0.001) and high-crown (*p* < 0.001) cores. Error bars denote standard deviation and letters indicate significant differences amongst core locations when all time points are pooled.

A one-way ANOVA revealed that preflooding CO_2_ fluxes were not significantly different amongst core locations (*p* = 0.109). Upon flooding, CO_2_ flux (averaged over days 1, 2, and 5) decreased by 43, 72, and 71% of that measured prior to flooding for low-crown, mid-crown, and high-crown cores, respectively (Appendix Table 1). For the remainder of the experiment, hourly CO_2_ fluxes from mid- and high-crown cores were not significantly different from each other, but were both significantly lower than the flux from low-crown cores (*p* < 0.001) (Fig. 3). A Pearson’s correlation test revealed that post-flood CO_2_ flux was negatively correlated with % soil compaction (*r* =  − 0.71, *p* = 0.004) with the highest CO_2_ fluxes measured from the least compacted (low-crown) cores.

### CH_4_ flux

**Figure 4:**
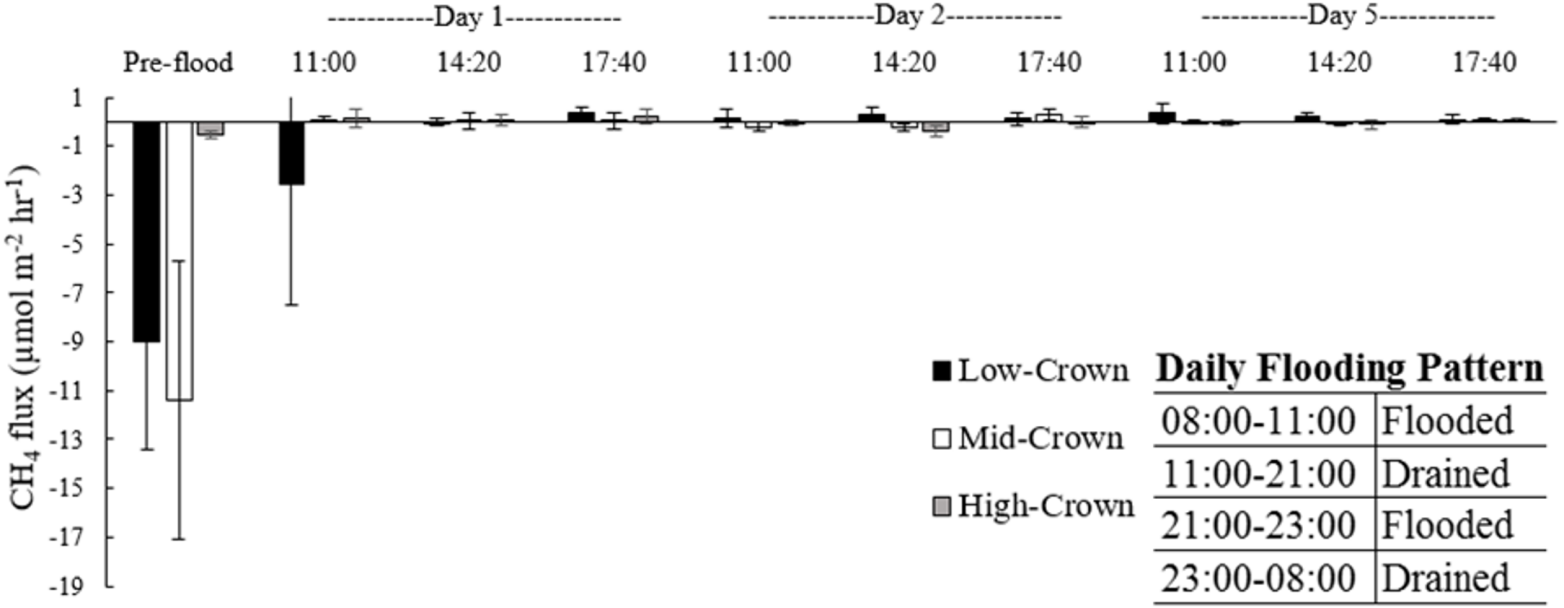
Mean CH_4_ flux from flooded soil cores collected from a drained salt marsh on the St. Lawrence estuary. After flooding CH_4_ flux remained low and did not differ significantly from zero (*p* = 0.733). Error bars denote standard deviation and letters indicate significant differences amongst core locations when all time points are pooled.

Prior to flooding CH_4_ fluxes were negative for all cores (Appendix Table 2). After flooding, mean CH_4_ fluxes increased, but were not significantly different from zero (*p* = 0.733). Except for the increase following the initial flooding there were no visible trends in CH_4_ flux throughout the experiment (Fig. 4).

### N_2_O flux

**Figure 5:**
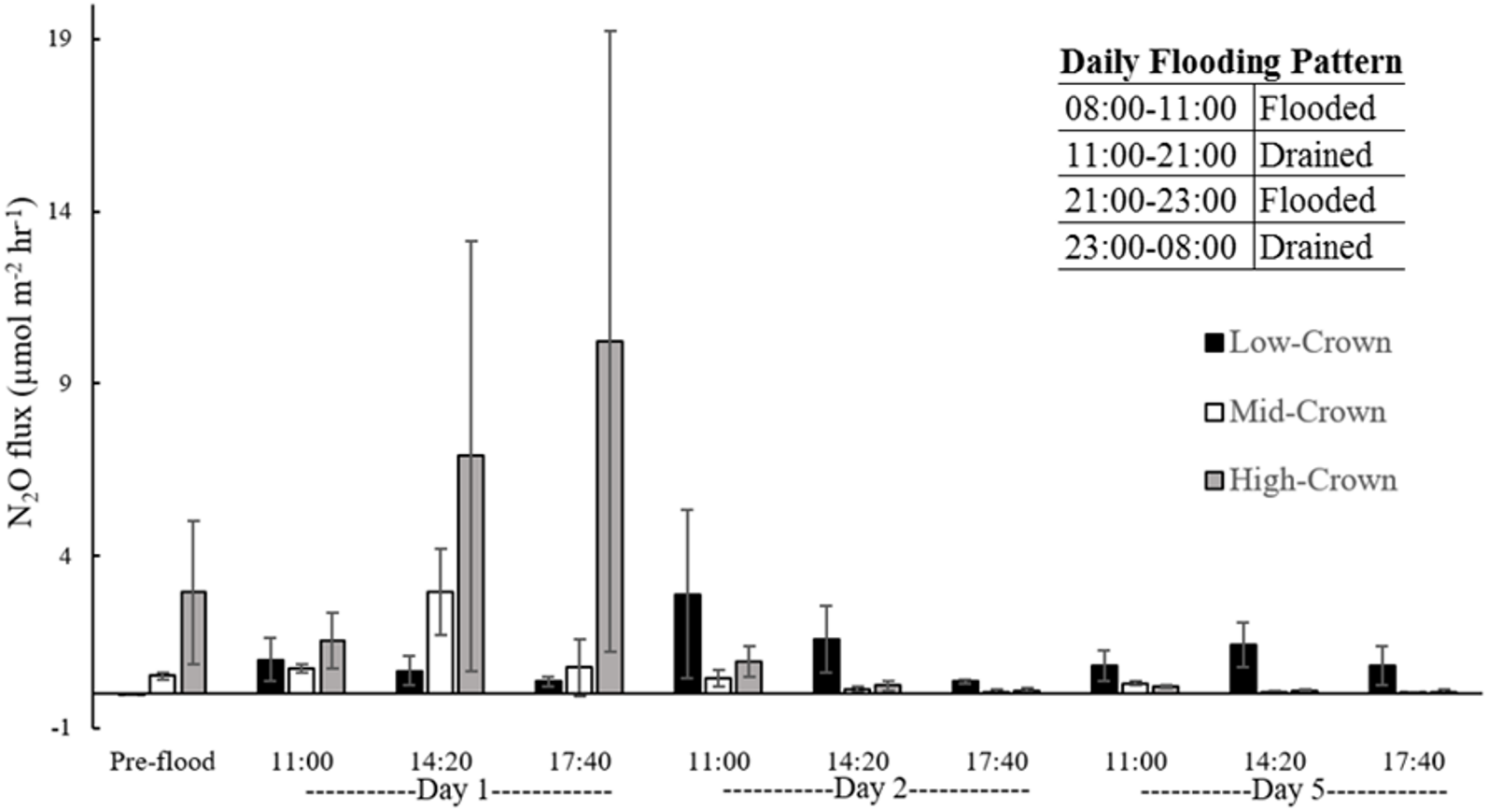
Mean N_2_O flux from flooded soil cores collected from a drained salt marsh on the St. Lawrence estuary. Daily N_2_O flux decreased significantly from day 1 to day 5 for mid-crown (*p* < 0.001) and high-crown (*p* = 0.001) cores. There was no significant difference in mean flux between days 1, 2, and 5 for low-crown cores (*p* = 0.117). Error bars denote standard deviation.

N_2_O fluxes were variable both amongst locations and individual cores (Appendix Table 3). A one-way ANOVA revealed that preflooding N_2_O fluxes varied significantly between different locations in the field (*p* = 0.012) with the highest fluxes measured in high-crown cores (mean flux of 2.94 µmol m^−2^hr^−1^) and small negative fluxes measured in low-crown cores (mean flux of −0.02 µmol m^−2^ hr^−1^) (Fig. 5).

The post-flooding pattern of N_2_O emission was similar for mid-crown and high-crown cores. After flooding, mean flux peaked at 10.22 µmol m^−2^ hr^−1^ in high crown cores on day 1 during the simulated low tide period (6 hr 40 min after the first flood event). Mean N_2_O flux from mid-crown cores peaked on day 1 of flooding at 2.96 µmol m^−2^ hr^−1^ during the simulated mid-tide period (4 hours after the first flood event). Peak fluxes were 540% and 350% higher than background levels for high-crown and mid-crown locations, respectively. Fluxes decreased significantly from day 1 at 17:40 hr to day 5 for mid-crown (*p* < 0.001) and high-crown (*p* = 0.001) cores; mean fluxes were near zero by the end of day 5.

Low-crown cores exhibited a different pattern and the highest hourly N_2_O flux was measured on day 2 immediately after the simulated tide receded. Unlike the mid and high-crown locations, positive fluxes from low-crown cores persisted above pre-flooding levels throughout the experiment. Despite reaching their highest level on day 2, there was no significant difference among mean N_2_O fluxes measured on days 1, 2, and 5 (*p* = 0.117). A Pearson’s correlation test revealed that post-flood N_2_O flux and soil N content were positively correlated (*r* = 0.186, *p* = 0.037) while background N_2_O flux and soil N content (NH_4_^+^ and NO_3_^−^ combined) were not (*p* = 0.256). In contrast, background N_2_O flux and % soil compaction were positively correlated (*r* = 0.642, *p* = 0.012) while post-flood N_2_O flux and % soil compaction were not (*p* = 0.295).

## Discussion

### CO_2_ flux

Mean CO_2_ fluxes from cores collected at the three different locations in the field significantly decreased with flooding. Soil physical properties which affect the diffusion and availability of oxygen can exhibit a strong control on decomposition rates and CO_2_ production. In well drained and aerated soils, oxygen diffusion is rapid and upland soils have O _2_concentrations near 21% up to depths of 1 m (Megonigal, Patrick & Faulkner, 1993). When soils are flooded, increasing the water filled pore space (WFPS) can slow O_2_ diffusion by a factor of 10^4^ compared with air (Megonigal, Hines & Visscher, 2004). As soils become saturated, some air-filled pore spaces can eventually become completely separated from gaseous exchange with atmosphere (Megonigal, Hines & Visscher, 2004). Increasing water content in the soil can thus slow aerobic decomposition and associated CO_2_ production (Hackney, 1987; Kristensen, Ahmed & Devol, 1995; Kirwan & Blum, 2011).

Changes in CO_2_ flux upon flooding in our experiment were consistent with other studies which have indicated that restoration of tidal flooding reduces decomposition in organic soils and prevents on-going C losses to the atmosphere (Pendleton et al., 2012). In another study carried out before and after the MR of a drained marsh in the UK cotton strip decomposition assays revealed rates of cotton tensile strength loss (CTSL), a proxy for decomposition rates, were greatly reduced following soil flooding (Blackwell, Hogan & Maltby, 2004).

Across the three locations sampled in the field, flooding resulted in a 62% decline in mean CO_2_ emissions. Extrapolated over 24 hr, this decrease is equivalent to a reduction in soil CO_2_ flux of 338 mmol CO_2_ m^−2^ day^−1^, nearly seven times the mean global C sequestration rate of 48 mmol CO_2_ m^−2^ day^−1^ reported by Chmura et al. (2003). In the short term, flooding dyked marsh soils could thus have a net cooling effect by reducing CO_2_-C losses and preventing the daily release of CO_2_ equivalent to one week of typical marsh C sequestration.

### CH_4_ flux

Negative CH_4_ fluxes occur when CH_4_ oxidation within the soil exceeds production and the soil acts as a sink (Conrad, 1996). Prior to flooding, CH_4_ fluxes were negative for all cores, with larger negative fluxes observed for mid-crown and low-crown cores. As soil CH_4_ flux is determined by a combination of gas diffusivity and methanotroph and methanogen activity (Von Fischer et al., 2009), differences in soil compaction may explain part of the variation in background fluxes between locations. Poor aeration and CH_4_ diffusion below the soil surface may thus have resulted in smaller CH_4_ uptake in the more compacted high-crown soils.

The negative pre-flooding fluxes for CH_4_ measured in our experiment range from −0.2 to −4.4 mg CH_4_ day^−1^ and are consistent with the net uptake of CH_4_ observed in many agricultural soils (Gattinger et al., 2014). Although soil flooding did not result in positive CH_4_ flux, CH_4_ uptake was significantly reduced. The magnitude of the change in flux should be approached with caution, however, as the background measurements were taken after the soils cores had been refrigerated and removed from the influence of rainfall or moisture. Given that differences in rainfall have been shown to shift agricultural soils from an annual sink to an annual source of CH_4_ (Chan & Parkin, 2001), refrigeration and removal of the cores from the influence of precipitation may thus have yielded larger negative fluxes than would have been expected in the field.

CH_4_ fluxes were negligible after flooding, presumably due to the abundance of sulfate remaining in the soil (Poffenbarger, Needelman & Megonigal, 2011). The highest hourly flux during the experiment (0.41 µmol m^−2^ hr^−1^) was measured during the simulated low-tide on the first day of flooding in the low-crown cores. Even if this maximum flux was maintained throughout the annual period when marsh soil is not frozen, the SGWP of CH_4_ emissions would offset <0.5% of the mean global C sequestration reported for salt marshes by Chmura et al. (2003). Thus, although the loss of the small CH_4_ sink is equivalent to an effective increase in the supply of CH_4_ to the atmosphere, the effect remains small when compared to the immediate reduction in CO2 emission as well as the future C sequestration once the marsh redevelops.

### N_2_O flux

Nitrous oxide (N_2_O) is produced primarily during three microbial processes in the soil nitrogen cycle: (1) the nitrification of ammonium (NH_4_^+^) to nitrate (NO_3_^−^), in which N_2_O is a non-obligate by-product, (2) the denitrification of NO_3_^−^ in which N_2_O is an obligate intermediate in the reduction of NO_3_ to nitrogen gas (N_2_), and (3) the dissimilatory reduction of NO_3_^−^ to NH_4_^+^ (DNRA) (Baggs, 2011; Butterbach-Bahl et al., 2013). These processes depend strongly on many inter-related factors including the availability of O_2_, NH_4_^+^ (for nitrification), NO_3_^−^ (for denitrification), and organic C (Megonigal, Hines & Visscher, 2004). N_2_O emission is thus expected to increase when NO_3_^−^-N and NH_4_^+^-N in the soil are higher (Signor & Cerri, 2013). The data from our experiment, however, showed a significant relationship between N_2_O flux and soil N content for post-flood measurements only; no significant relationship was observed between pre-flood N_2_O flux and soil N content.

Differences in soil compaction offer one possible explanation for the variation in pre-flood fluxes among cores collected from the three different locations in the farm field. Denitrification occurs when the consumption of O_2_ in the soil by plant roots and microorganisms exceeds the rate of replenishment from the atmosphere and creates anoxic microsites in the soil (Smith et al., 2003). Oxygen is the energetically preferred electron acceptor in denitrifying bacteria and if present will result in the suppression of denitrification enzyme synthesis (Megonigal, Hines & Visscher, 2004). Still, as NO_3_^−^ readily accepts electrons in the absence of O_2_, denitrification can begin rapidly once anaerobic conditions develop (Smith et al., 2003). Compaction decreases gas diffusivity and oxygen delivery to the soil (Smith et al., 2003) and may thus increase the proportion of anaerobic microsites in which N_2_O is produced (Megonigal, Hines & Visscher, 2004).

Field experiments and models have shown higher N_2_O emissions in compacted soil (Ball, Scott & Parker, 1999). Sitaula, Hansen & Sitaula JIB (2000) observed, however, that increases in N_2_O emission from soil compaction in the field became non-significant when soil was broken up and sieved. As they found the effect of compaction on N_2_O flux to be reversible, Sitaula, Hansen & Sitaula JIB (2000) suggest that it was the physical effects of compaction that drove the increase in N_2_O flux rather than a permanent increase in the biological potential for N_2_O production (Sitaula, Hansen & Sitaula JIB, 2000). This is consistent with the St. Lawrence cores for which % soil compaction was positively correlated with background N_2_O flux. It should be noted that although mechanical compaction occurred during core collection, it is likely that the soil in the mid-crown and high-crown locations was already more compacted *in situ*. In the low-crown location, piping was easily driven into the soil whereas considerable resistance was encountered when the same force was applied by the hydraulic borer in the mid and high-crown locations. The effects of compaction are thus likely attributable to both pre-existing compaction within the farm soils as well as the additional compaction that occurred during sampling.

After 5 days of flooding only low-crown cores showed positive N_2_O flux with the more compacted soils in the mid-crown and high-crown cores showing fluxes near zero. Given that agricultural lands tend to have higher N_2_O emissions (Meurer et al., 2016), these results suggest that in highly compacted soils, which are frequently encountered in agricultural settings due to tractor and machinery traffic (Jabro et al., 2014), returning tidal flooding may in fact quickly transform a former source of N_2_O into a sink. For the less compacted low-crown soils, if the mean daily flux of 1.01 µmol m^−2^ hr^−1^ were to persist for the entire period of the year during which soils are not frozen, the SGWP of the additional N_2_O emissions would offset <10% of the mean global C sequestration reported for salt marshes by Chmura et al. (2003). The recovering marsh would thus maintain a global cooling potential.

### Significance of combined GHG fluxes in terms of global warming potential

After flooding, the 5-day mean SGWP fell by 72, 70, and 71% for low-crown, mid-crown, and high-crown soil cores, respectively (Table 3).

**Table 3:** Mean sustained global warming potentials (Neubauer & Megonigal, 2015) for the GHG fluxes (mmol CO_2_ eq m^−2^ day^−1^) from flooded dykeland soil cores.

		N_2_O	CH_4_	CO_2_	Total SGWP
Background (pre-flood)	Low-Crown	−0.19	−43.93	464	420
	Mid-Crown	0.15	−55.47	600	545
	High-Crown	0.79	−2.65	534	532.9
5 day mean^a^ (post flood)	Low-Crown	7.64	−0.51	256	264
	Mid-Crown	2.78	−0.10	160	163
	High-Crown	9.44	−0.31	146	155

**Notes.**

a No measurements were made on days 3 and 4. Gas flux on days 3 and 4 were thus assumed to be equivalent to the mean of fluxes measured on days 2 and 5.

The substantial cooling effect associated with the reduction in CO_2_ flux far exceeded the warming potential associated with the small increases in N_2_O and CH_4_ flux. Restoring tidal flooding to the historically dyked soils thus had an immediate net cooling effect and does not appear to pose a risk of offsetting the global cooling potential of the C that would be sequestered as the salt marsh recovered. Going forward, the successfully restored marsh would then be expected to resume new soil C storage at rates typical for the region. In addition, although we did not consider the C sequestration associated with the growth of farm vegetation, salt marsh production is comparable to crop production (Reidenbaugh, 1983; USDA, 2014).

## Conclusions

Previous studies have raised concerns that re-flooding drained salt marsh soils may temporarily create conditions favorable for denitrification and N_2_O production. Due to its high SGWP, even small fluxes of N_2_O can offset a portion of the blue carbon benefit associated with a marsh restoration project. Here we show that despite small increases in CH_4_ and N_2_O flux following flooding, the accompanying large decline in CO_2_ emission suggests that re-flooding has a strong and immediate net cooling effect. The daily SGWP of both CH_4_ and N_2_O were very small when compared with the decline in CO_2_ and CH_4_ and N_2_O contributed <5% of the total mean daily SGWP when expressed in terms of CO_2_ eq.

The use of soil cores allowed us to measure emissions at the exact moment of reflooding. Further monitoring beyond the first week of flooding is still warranted to confirm the persistence of low N_2_O flux, particularly at times such as spring thaw when flux is expected to be higher. In addition the N-content of the soils tested in this experiment were low compared with those measured in other agricultural soils and further study of N_2_O emissions following restoration may be necessary for areas which have been heavily fertilized. Still, our findings suggest that N_2_O emissions following re-flooding are likely to be minimal in non-fertilized dykelands and provide further support for the effectiveness of marsh restoration as a climate mitigation strategy.

##  Supplemental Information

10.7717/peerj.5659/supp-1 Data S1 Raw data and supplementary material

## Additional Information and Declarations

### Data Availability

The following information was supplied regarding data availability: The raw data are provided in Data S1.
